# Moderating and mediating effects of resilience between childhood trauma and psychotic-like experiences among college students

**DOI:** 10.1186/s12888-024-05719-x

**Published:** 2024-04-12

**Authors:** Heqiong Hu, Chunping Chen, Bingna Xu, Dongfang Wang

**Affiliations:** 1 School of New Media Technology, Hunan Mass Media Vocational and Technical College, Changsha, China; 2 Hunan Academy of Education Sciences, Changsha, China; 3 Institute of Education, Xiamen University, Xiamen, China; 4 School of Psychology, Centre for Studies of Psychological Applications, Guangdong Key Laboratory of Mental Health and Cognitive Science, Ministry of Education Key Laboratory of Brain Cognition and Educational Science, South China Normal University, Guangzhou, China

**Keywords:** Childhood trauma, Resilience, Psychotic-like experiences, Mediation, Moderation, College students

## Abstract

**Background:**

Extensive literature revealed that childhood trauma serves as a significant risk factor for developing psychotic-like experiences (PLEs) among the general population. Resilience has been regarded as a protective factor against PLEs. However, it remains unclear what role resilience plays in the relationship between childhood trauma and PLEs.

**Methods:**

A total of 4302 college students completed the web-based survey in January 2021. Participants completed self-report measures of sample characteristics variables, childhood trauma, and PLEs. Moderation and mediation analyses were adopted to examine the associations linking childhood trauma, resilience, and PLEs.

**Results:**

PLEs were positively associated with childhood trauma while negatively associated with resilience. Resilience played a partially mediating role in the relationship between childhood trauma and PLEs. Additionally, resilience moderated the association of childhood trauma with PLEs.

**Conclusions:**

These findings indicated that resilience plays a crucial role in mediating the relationship between childhood trauma and PLEs, suggesting the potential clinical implication of enhancing resilience for the prevention and intervention of PLEs among college students.

## Introduction

Psychotic-like experiences (PLEs) are conceptualized as the resemblance of positive symptoms of psychosis in the absence of a full-blown psychotic disorder and mostly transient in nature [1]. PLEs are commonly observed in both adolescents and young adults, while the prevalence rate reported is highly variable because of different measures and ways of constructing definitions. For example, Mamah et al. using the Washington Early Recognition Center Affectivity and Psychosis (pWERCAP) Screen reported that 72% of the adolescents and young adults in Kenya experienced at least one PLE within the past year [2]. In contrast, Adewuya et al. found that 10.5% of adolescents in Lagos had clinically significant PLEs, based on the 16-item version of the Prodromal Questionnaire [3]. In the Chinese context, according to the 8-item Positive Subscale of the Community Assessment of Psychic-Experiences (CAPE-P8), Sun et al. revealed that 20.7% of adolescents and young adults suffered from frequent PLEs during their lifetime [4]. Using the same instrument, namely CAPE-P8, a recent study showed that nearly half of the junior and senior high school students (49.3%) had at least one episode of PLE within a month and 15.4% of adolescents had frequent PLEs [5]. In general, the prevalence rate of PLEs increased from age 13 to 24, with a substantial peak during late adolescence [6]. Meanwhile, PLEs are considered as a predictive indicator of many mental health illnesses. PLEs are associated with an increased risk of mental disorders, as well as significant impairments in social functioning [1]. For instance, PLEs in early life are related to an improved chance of later psychotic disorders [7], mood disorder [8], self-harm or suicidal behavior [9], and increased mental health service use [10]. Given the prevalence of PLEs and their deleterious impact on future individual well-being, it is necessary to investigate influential factors for PLEs, particularly in late adolescence (e.g., college students), to provide crucial information for early mental health interventions.

The pathogenesis of PLEs is a complex process influenced by various factors, including environmental and genetic factors [11, 12]. Childhood trauma is proven to be one of the environmental factors that can contribute to PLEs [13, 14]. Childhood trauma encompasses a range of adverse events occurring in childhood, including but not limited to neglect and abuse, and may lead to mental health problems across the lifespan [15]. Substantial clinical and epidemiological studies offered compelling evidence that those who have experienced childhood trauma are more likely to sustain PLEs [4, 16, 17]. Research has also indicated a positive relationship between childhood trauma and PLEs among college students [18–20].

Undoubtedly, resilience is a crucial protective factor for an individual’s mental health and has been given various meanings by scholars in previous studies. For some, resilience is considered as an outcome [21], a process [22], and a modifiable personal quality [23]. Given this, previous studies on childhood trauma and psychopathology have raised a host of modalities (i.e., mediating or moderating) through which resilience is related to poor mental health. This view coincides with the results of a recent meta-analysis, which showed that resilience is both a mediator and moderator of the correlation between childhood trauma and depression [24]. Specifically, when resilience is understood as an intermediate process, wherein an individual presents a positive adaptation to stress, crisis, and adversity [25], it may be the link between childhood trauma and psychological problems. For instance, several studies indicated that resilience mediated the relationship between childhood trauma and mental health problems [26, 27]. While in some studies, resilience was also found to act as a moderator to alleviate the negative effects of childhood trauma on individual mental health [28, 29]. From the perspective of the risk-protective theory, resilience can play a buffering role in psychological distress to compensate for stressors [30]. To conclude, current research on the role of resilience in the association between childhood trauma and PLEs is limited, particularly among adolescents and young adults.

Given the above, college students were investigated in this study to explore the associations linking childhood trauma, resilience, and PLEs. Our major hypotheses are: (1) childhood trauma is positively significantly associated with PLEs among college students; (2) resilience mediates the childhood trauma-PLEs link; (3) resilience moderates the childhood trauma-PLEs association.

## Methods

### Study design and participants

In January 2021, we collected data via a web-based survey at Shanxi Technology and Business University in Taiyuan, Shanxi Province, China. Taiyuan, a representative city in central China, is a prominent industrial hub primarily focused on energy and heavy chemicals. Prior to the survey, we dispatched electronic invitation letters to students through WeChat with the assistance of counselors from each department. These invitation letters conveyed the purpose of this survey and assured participants of the confidentiality of the survey results. Informed consent was obtained from all participants (or their guardians if age < 18) before the investigation. Data were collected through ‘Questionnaire Star’ system (i.e., a domestic platform specializing in the collection of online questionnaires; www.wjx.cn) with a quick response (QR) code. Students scanned the QR code of the questionnaires through their mobile phones to complete the online survey. The survey also followed the principle of voluntary participation. Participants were allowed to withdraw freely if they experienced discomfort during the test. During the survey, a free psychological hotline was available to provide assistance to participants who needed it. The investigation was carried out in accordance with the Helsinki Declaration as revised in 1989 and approved by the Ethics Committees of South China Normal University.

In this study, inclusion criteria for participation included: (a) upload the informed consent form with signature; (b) response time for online survey was above 5 min; and (c) have no current significant physical disease or history of psychiatric illness. Finally, a total of 4302 college students were involved in the study. A detailed description of the present study design has been described previously [31].

### Measures

#### Childhood trauma

Childhood trauma before the age of 16 was measured by the Childhood Trauma Questionnaire (CTQ) [32]. It consists of 28 items, clustering into 5 dimensions: emotional abuse (EA), physical abuse (PA), sexual abuse (SA), emotional neglect (EN), and physical neglect (PN). Each item is rated on a 5-point Likert scale, from 1 (never) to 5 (always), with a higher score reflecting a greater number of childhood traumas experienced. The Chinese version of CTQ has satisfactory psychometric properties [33]. In this sample, Cronbach’s α was 0.82.

#### Resilience

Resilience level was assessed by 10-item Connor-Davidson Resilience Scale (CD-RISC-10) [34]. Each item was rated on a 4-point Likert scale, from 0 (not true at all) to 4 (true nearly all of the time), and a higher total score suggested a greater level of resilience. The Chinese version of CD-RISC-10 has good reliability and validity [35, 36], and Cronbach’s α was 0.97 in this study.

#### PLEs

The frequency of PLEs was measured by the Chinese version of CAPE-P8 [37, 38]. The CAPE-P8 is extracted from the 42-item CAPE [39] and assesses two dimensions of delusional experiences (DEs) and hallucinatory experiences (HEs). Four response options are available on a scale of 1 (never) to 4 (nearly always). A higher score means more frequent PLEs. The presence of PLEs was defined as having “sometimes”, “often” or “nearly always” on one or more items [5], and Cronbach’s α was 0.83 in this study.

#### Sample characteristic variables

Sample characteristic variables included sex, grade, age, ethnicity, parental marital status, family income, parents’ education, and single-child status.

### Statistical analysis

Statistical analyses were carried out with SPSS 23.0. The Mann–Whitney U-test (for continuous variables) and chi-square test (for categorical variables) were used to compare the sample characteristic variables, CTQ, and CD-RISC-10 score between the No-PLEs and PLEs groups. The Spearman correlation analysis was carried out to assess the bivariate correlations between CTQ, CD-RISC-10, and CAPE-P8 scores. Potential multicollinearity among all variables was assessed by a variance inflation factor (VIF) [40]. Before model analysis, the exploratory factor analysis examines common method bias [41]. The PROCESS macro v3.3 was applied to examine the hypothetical mediation and moderation model. According to Hayes’ suggestions [42], we employed Model 1 to investigate the moderating role of resilience between childhood trauma and PLEs. All continuous variables were standardized. We entered childhood trauma as an independent variable, resilience as the moderator, and PLEs as the dependent variable. Previous research indicated that PLEs are associated with numerous sample characteristics, such as ethnic minority status [43], parental marital status [5], and family income [5]. Parents’ education [44] and single-child status [45] are also found to be associated with childhood trauma. Thus, we controlled for sample characteristic variables in model testing. The 5000 bootstrapping resamples method could attain 95% confidence intervals (95% CI) to estimate both direct and indirect effects simultaneously. Similarly, we set resilience as the mediator and explored the mediating effect of resilience using Model 4.

## Results

### Descriptive statistics

The study included 4302 college students, of which 1780 were male (41.1%). The age of the students ranged from 16 to 25 years; the mean (SD) age was 19.92 (± 1.42) years. The vast majority of students (99.4%, *N* = 4277) are of Han ethnicity and 25.7% were from single-child families. Other sample characteristics of participants are listed in Table 1.

**Table 1:** Sample characteristics of 4302 college students

Characteristics	Total	No-PLE (*N* = 2488)	PLEs ^c^ (*N* = 1814)	P^d^
Sex				0.259
Male	1780(41.4)	1011(40.6)	769(42.4)	
Female	2522(58.6)	1477(59.4)	1045(57.6)	
Age [year, Mean (SD)]	19.92(1.42)	20.02(1.44)	19.79(1.39)	< 0.001
Grade				< 0.001
Freshman	1876(43.6)	1027(41.3)	849(46.8)	
Sophomore	1133(26.3)	581(23.4)	552(30.4)	
Junior	1044(24.3)	715(28.7)	329(18.2)	
Senior	249(5.8)	165(6.6)	84(4.6)	
Ethnicity				0.550
Han ^a^	4277(99.4)	2475(99.5)	1802(99.3)	
Others	25(0.6)	13(0.5)	12(0.7)	
Parental marital status				0.077
Married	4005(93.1)	2331(93.7)	1674(92.3)	
Not current married ^b^	297(6.9)	157(6.3)	140(7.7)	
Family income (monthly), RMB				0.664
< 3000	2419(56.2)	1386(55.7)	1033(56.9)	
3000 ∼ 5000	1153(26.8)	679(27.3)	474(26.2)	
> 5000	730(17.0)	423(17.0)	307(16.9)	
Father’s education				0.229
Junior high school or below	2831(65.8)	1637(65.8)	1194(65.8)	
Senior high school	914(21.2)	513(20.6)	401(22.1)	
College or above	557(12.9)	338(13.6)	219(12.1)	
Mother’s education				0.589
Junior high school or below	3009(69.9)	1725(69.3)	1284(70.8)	
Senior high school	846(19.7)	500(20.1)	346(19.1)	
College or above	447(10.4)	263(10.6)	184(10.1)	
Single-child status				0.148
Yes	1107(25.7)	661(26.6)	446(24.6)	
No	3195(74.3)	1827(73.4)	1368(75.4)	
CTQ score [Mean (SD)]	35.14(12.77)	33.35(11.67)	37.59(13.78)	< 0.001
EA score [Mean (SD)]	6.23(2.58)	5.79(2.21)	6.82(2.91)	< 0.001
PA score [Mean (SD)]	5.65(2.46)	5.42(2.15)	5.97(2.80)	< 0.001
SA score [Mean (SD)]	5.54(2.39)	5.34(2.10)	5.82(2.71)	< 0.001
EN score [Mean (SD)]	9.69(6.29)	9.17(6.26)	10.40(6.26)	< 0.001
PN score [Mean (SD)]	8.02(3.43)	7.62(3.25)	8.58(3.58)	< 0.001
CD-RISC-10 score [Mean (SD)]	30.66(7.92)	32.22(7.50)	28.52(7.98)	< 0.001
CAPE-P8 score [Mean (SD)]	9.20(2.20)	8.00(0.00)	10.85(2.60)	< 0.001
DEs score [Mean (SD)]	2.15(0.51)	6.00(0.00)	8.49(2.13)	< 0.001
HEs score [Mean (SD)]	7.05(1.85)	2.00(0.00)	2.36(0.74)	< 0.001

*Abbreviations* CTQ = Childhood Trauma Questionnaire; EA = emotional abuse, PA = physical abuse, SA = sexual abuse, EN = emotional neglect, PN = physical neglect, CD-RISC-10 = 10-item Connor-Davidson Resilience Scale; PLEs = psychotic-like experiences; DEs = delusional experiences, HEs = hallucinatory experiences

^a^ Han is the major ethnic group in China

^b^ Not current married included separated, divorced and widowed

^c^ PLEs, Psychotic-like experiences calculated using the CAPE-P8, with one or more items were selected 2 (sometime) and above

^d^ Chi-squared tests were used for categorical variables; t test was used for continuous variables

In this sample, 42.2% of participants (*N* = 1814) had at least one PLE in the past month. This part of the sample is defined as PLEs, and the rest is the non-PLE group. The sample characteristics of the PLEs group and non-PLE group are also presented in Table 1. The two groups were significantly different in age (t = 5.19, *p* < 0.001) and grade (χ^2^ = 83.14, *p* < 0.001). Compared with the non-PLE group, PLEs group tended to have higher CTQ scores and five subscale (i.e., EA, PA, SA, EN, and PN) scores while with lower CD-RISC-10 scores. Moreover, only 4.8% of the sample (*N* = 209) sustained frequent (“often” or “nearly always”) PLEs.

### Associations among main study variables

PLEs were positively associated with childhood trauma (*r* = 0.25, *p* < 0.001) while negatively associated with resilience (*r* = -0.29, *p* < 0.001), age (*r* = -0.07, *p* < 0.001), and grade (*r* = -0.09, *p* < 0.001). Resilience had a negative relationship with childhood trauma (*r* = -0.28, *p* < 0.001). Moreover, the VIF of all variables was 1.09 and below, denoting a low possibility of multicollinearity.

### Testing for the moderating effect of resilience

**Fig. 1:**
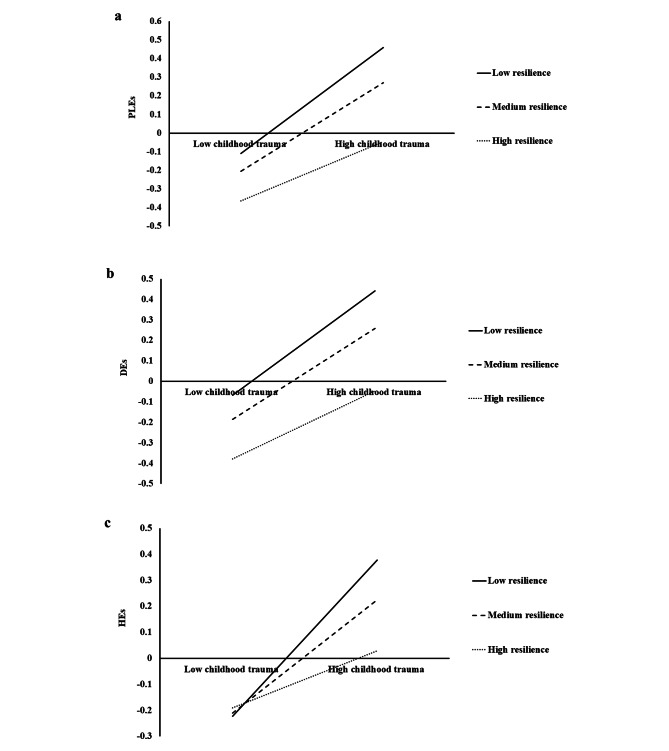
The interaction between childhood trauma and resilience in predicting PLEs shows childhood trauma was associated with PLEs weaker with higher resilience; PLEs = psychotic-like experiences; DEs = delusional experiences, HEs = hallucinatory experiences; Fig. 1a denotes PLEs as the outcome variable, Fig. 1b denotes DEs as the outcome variable, and Fig. 1c denotes HEs as the outcome variable

An exploratory factor analysis found 6 factors with eigenvalues more than 1 and the first factor accounted for 12.36% of the total variance, indicating that common method variance was not of great concern in this study. The moderating effect was examined, with childhood trauma entered as the predictor, resilience as the moderator, and PLEs as the outcome. Sample characteristics variables were included in the analyses as covariates. As shown in Table 2, both childhood trauma (b = 0.25, 95% CI = 0.22 ∼ 0.28) and resilience (b = -0.18, 95% CI = -0.21 ∼ -0.15) had a main effect on PLEs. The interaction of childhood trauma and resilience was significantly associated with PLEs (b =-0.07, 95% CI = -0.01 ∼ -0.04), indicating that resilience moderated the relationship between childhood trauma and PLEs. In other words, the effect of childhood trauma on PLEs significantly varied at levels of resilience. Further, simple slope analyses found a significant positive association between childhood trauma and PLEs was weaker at higher (b = 0.30, 95% CI = 0.27 ∼ 0.34) than medium (b = 0.25, 95% CI = 0.22 ∼ 0.28) and lower (b = 0.17, 95% CI = 0.13 ∼ 0.21) levels of resilience (see Fig. 1a). These findings proved that high resilience may buffer the relationship between childhood trauma and PLEs. Similar protective effect of resilience is also found in the relationship between childhood trauma and Des (see Fig. 1b)/HEs (see Fig. 1c).

**Table 2:** Regression results of the moderation of resilience in the relationships between childhood trauma and PLEs ^#^

Outcome	Predictors	b	t	p	95% CI
PLEs	CT	0.25	16.47	< 0.001	0.22,0.28
	Resilience	-0.18	-10.95	< 0.001	-0.21,-0.15
	CT×Resilience	-0.07	-5.20	< 0.001	-0.10,-0.04
	*Simple slope analysis*				
	Low resilience	0.30	16.74	< 0.001	0.27,0.34
	Medium resilience	0.25	16.84	< 0.001	0.22,0.28
	High resilience	0.17	7.87	< 0.001	0.13,0.21
DEs	CT	0.23	15.34	< 0.001	0.20,0.26
	Resilience	-0.19	-11.58	< 0.001	-0.22,-0.16
	CT×Resilience	-0.05	-3.64	< 0.001	-0.07,-0.02
	*Simple slope analysis*				
	Low resilience	0.27	14.88	< 0.001	0.24,0.31
	Medium resilience	0.24	15.60	< 0.001	0.21,0.27
	High resilience	0.18	8.17	< 0.001	0.13,0.22
HEs	CT	0.22	14.44	< 0.001	0.19,0.25
	Resilience	-0.08	-4.52	< 0.001	-0.11,-0.04
	CT×Resilience	-0.12	-8.94	< 0.001	-0.14,-0.09
	*Simple slope analysis*				
	Low resilience	0.32	17.25	< 0.001	0.28,0.36
	Medium resilience	0.23	15.07	< 0.001	0.20,0.26
	High resilience	0.09	3.87	< 0.001	0.04,0.13

*Abbreviations* CT = Childhood trauma; PLEs = psychotic-like experiences; DEs = delusional experiences, HEs = hallucinatory experiences; CI = Confidence interval

^#^ Adjusting for sex, grade, age, ethnicity, parental marital status, family income, parents’ education, and single child status

### Testing for the mediating effect of resilience

**Fig. 2:**
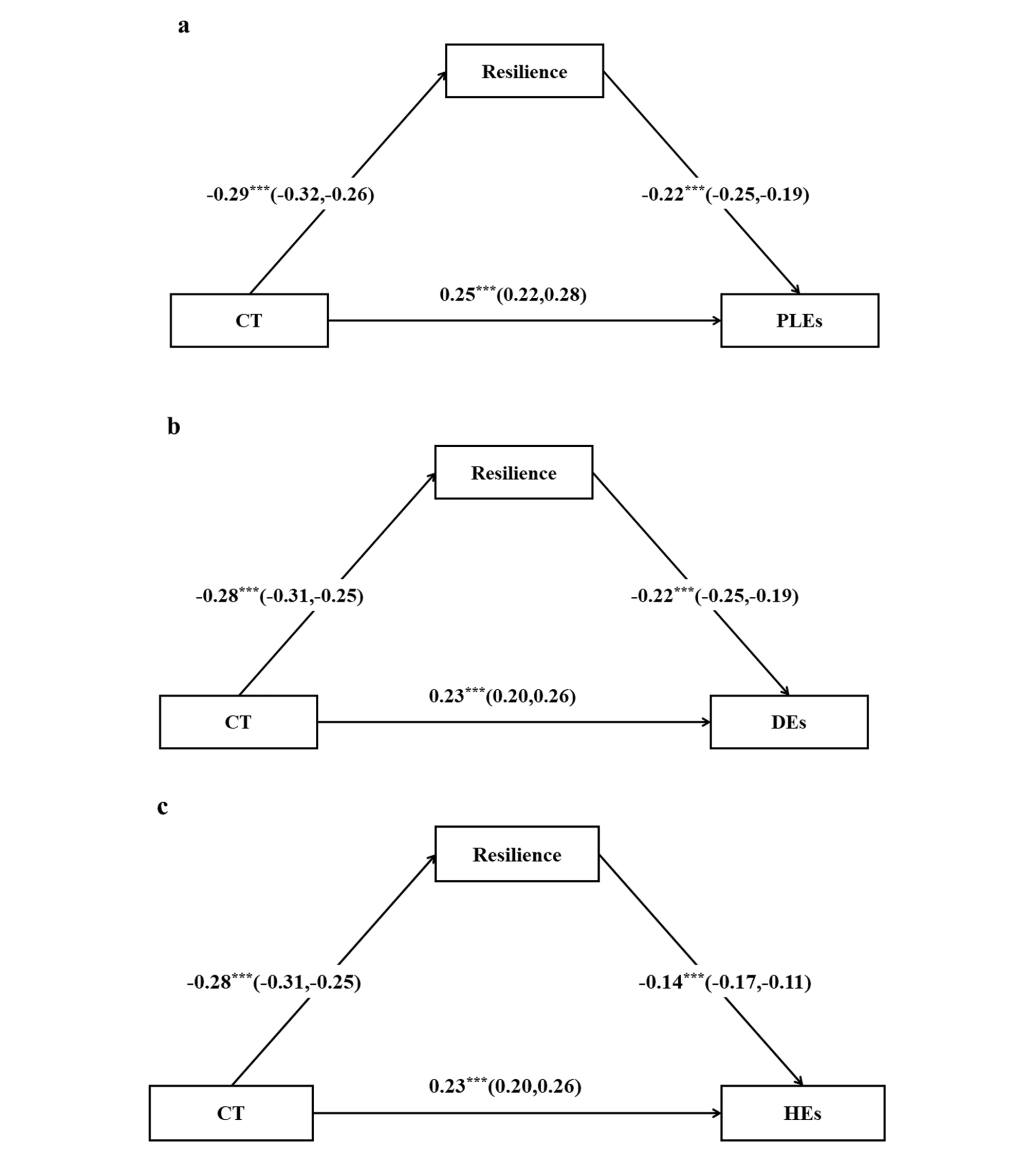
Standardized path coefficients (95% confidence intervals) for the mediating model for PLEs. Abbreviations: CT = Childhood trauma; PLEs = psychotic-like experiences; DEs = delusional experiences, HEs = hallucinatory experiences; Fig. 2a denotes PLEs as the outcome variable, Fig. 2b denotes DEs as the outcome variable, and Fig. 2c denotes HEs as the outcome variable. Adjusted for sex, grade, age, ethnicity, parental marital status, family income, parents’ education, and single child status; ^***^p < 0.001

Figure 2a depicts the standardized regression results of mediation to test the significance of the effects of childhood trauma on PLEs through resilience, after controlling for sample characteristics variables. Childhood trauma had a significant negative effect on resilience (b = -0.29, 95% CI = -0.32 ∼ -0.26), as well as a significant positive effect on PLEs (b = 0.25, 95% CI = 0.22 ∼ 0.28). While resilience negatively predicted PLEs (b = -0.22, 95% CI = -0.26 ∼ -0.19). Therefore, resilience significantly and partially mediated the relation between childhood trauma and PLEs (indirect effect = 0.06, 95% CI = 0.05 ∼ 0.08). The model explained 8.6% variances in resilience and 14.9% variances in PLEs. Similar mediating effect of resilience is also found in the relationship between childhood trauma and Des (indirect effect = 0.06, 95% CI = 0.05 ∼ 0.08, see Fig. 2b)/HEs (indirect effect = 0.04, 95% CI = 0.03 ∼ 0.05, see Fig. 2c).

## Discussion

The present study aimed to deepen our understanding of how childhood trauma may be linked to PLEs by testing the mediating and moderating roles of resilience with a sample of college students. Our findings supported the hypothesis, indicating that resilience might mediate the effect of childhood trauma on PLEs, and also moderate the relationship of childhood trauma with PLEs.

In this sample, 42.2% of college students have had at least one PLE in the past month, which is lower than the finding with the same measure of Chinese junior and senior high school students (49.3%) [5]. Only 4.8% of college students have frequent PLEs. PLEs are infrequent for most adolescents who experienced them [46, 47]. In addition, PLEs were negatively associated with age and grade. In fact, 75 ∼ 90% of PLEs are temporary in adolescents, resolving or even disappearing with age, and only a small proportion would develop into persistent conditions [1].

Consistent with previous studies [18–20], childhood trauma was positively associated with PLEs. This relationship can be explained by several theories, including the genetic predisposition hypothesis, stress-vulnerability model, and attachment theory [48]. The psychosis proneness-persistence-impairment model [1] indicated that genetic factors may interact with environmental risk during childhood (e.g., early trauma), which could result in traits such as biological and psychological sensitization. These traits can potentially contribute to the persistence of PLEs with adverse outcomes. Additionally, structural and functional brain alterations and basic neurocognitive deficits caused by childhood trauma led to increased vulnerability to PLEs [13, 49–51].

In line with previous studies [20, 52], resilience was negatively associated with PLEs. Resilience has been regarded as a protective factor against poor mental health; better resilience facilitates remission of PLEs [53]. Our results indicated that resilience moderates the association between childhood trauma and PLEs, which is in line with previous studies. For instance, Dale et al. concluded that resilience has a moderating effect between childhood sexual abuse and depression among women with and at risk for HIV [54]. Sleijpen et al. also found a moderating effect of resilience between potentially traumatic events and mental health problems and life satisfaction in refugees and adolescents [55]. The finding also supported the risk-protective theory [30] that resilience can paly a buffering role in mental health to compensate for negative stressors. The results of simple slope analysis also suggested that the effect of childhood trauma on PLEs significantly varied at levels of resilience. More specifically, these findings revealed that having experience of childhood trauma, college students with a higher level of resilience developed less frequency of PLEs, while those with a lower level of resilience developed a greater frequency of PLEs. In brief, with higher resilience, the association between childhood trauma and PLEs became weaker.

Our results also proved that resilience was a partial mediator between childhood trauma and PLEs. The results corresponded to previous studies that resilience played a mediating role between early traumatic life events and PLEs in young adults [56]. In Ungar’ s opinion, resilience is the ability of adolescents to navigate towards health-maintaining resources they needed in the context of adversity [57]. Higher resilience may enable people to leverage their personal positive resources [58] and successfully adapt to adversity [59]. Individuals who are better able to cope with adversity or stressful events exhibit fewer psychological problems [60]. By contrast, individuals who have experienced major childhood trauma may change their attitude toward the world (i.e., pessimistic, helpless, and hopeless), unable to adapt to adversity well and may develop many mental disorders [61].

This study also showed signiciatnt mediating and moderating effects of resilience both in the relationship between childhood trauma and DEs/ HEs. Interestingly, our findings suggest that resilience paly a slightly stronger mediating role on childhood trauma - DEs link, while paly a stronger moderating role on childhood trauma - HEs associatioin. This result may reflect a greater direct effect of resilience on DEs, but for HEs, resilience has a primarily indirect effect by moderating childhood trauma. However, in one of our previous studies, we showed that the moderating role of resilience in sleep distrbance and DEs see little difference from that between sleep distrbance and HEs [52]. Further research is therefore necessary to explore the role of resilience on DEs and HEs.

This study has several limitations that need to be noted. First, all the measures used in the current study were self-reported, which may lead to recall bias of history of childhood trauma and frequency of PLEs. Second, this study is based on a cross-sectional survey, which may limit the inference of causality. Moreover, the current study examined the effects of overall childhood trauma on PLEs, without differentiating types of trauma. Different subtypes of childhood trauma may have different impacts on PLEs [62], which should be explored in the following studies by recruiting more participants. Third, our data were collected through online sources, exclusively focusing on students from a single university. Consequently, caution is advised when interpreting the accuracy and representativeness of the data. Moreover, it shall be noted that our data might be influenced by the COVID-19 pandemic since its associated stresses may increase the severity of PLEs [20, 63]. Finally, several important confounder factors that may affect the study findings, such as current negative life events and depressive symptoms, have not been considered.

## Conclusion

Childhood trauma and impaired resilience were associated with elevated risk of PLEs among college students. Resilience moderated the association of childhood trauma with PLEs, as well as partially mediated the effect of childhood trauma on PLEs. The observed moderating and mediating effects of resilience in this study emphasized the need to develop and implement prevention and interventions for PLEs through resilience enhancement training. These findings draw attention to the potential roles of educators and clinicians in considering and adopting measures to promote adolescents’ resilience.

## Data Availability

The datasets used and/or analyzed during the current study are available from the corresponding author (Dr. DW) on reasonable request.
